# Personalizing Treatment for Pancreatic Ductal Adenocarcinoma: The Emerging Role of Minimal Residual Disease in Perioperative Decision-Making

**DOI:** 10.3390/cancers18010094

**Published:** 2025-12-27

**Authors:** Charalampos Theocharopoulos, Nikolaos Machairas, Ioannis A. Ziogas, Benedetto Mungo, Marco Del Chiaro, Georgios K. Glantzounis, Richard Schulick, Georgios C. Sotiropoulos

**Affiliations:** 1 Department of Surgery, University of Colorado Anschutz Medical Campus, Aurora, CO 80045, USA; ioannis.ziogas@cuanschutz.edu (I.A.Z.); benedetto.mungo@cuanschutz.edu (B.M.); marco.delchiaro@cuanschutz.edu (M.D.C.); richard.schulick@cuanschutz.edu (R.S.); 2 2nd Department of Propaedeutic Surgery, National and Kapodistrian University of Athens, 11527 Athens, Greece; nmachair@gmail.com; 3 Department of Liver Transplantation and Hepatobiliary Surgery, National and Kapodistrian University of Athens, 11527 Athens, Greece; gsotirop@yahoo.com; 4 Department of Surgery, University Hospital of Ioannina, 45500 Ioannina, Greece; gglantzounis@uoi.gr

**Keywords:** pancreatic ductal adenocarcinoma, minimal residual disease, circulating tumor cells, circulating tumor DNA, adjuvant treatment, neoadjuvant treatment, pancreatoduodenectomy

## Abstract

Pancreatic ductal adenocarcinoma (PDAC) is a highly aggressive malignancy with poor long-term survival despite advances in surgical techniques, systemic therapies, and perioperative management. High rates of systemic recurrence following curative-intent resection suggest that many patients harbor minimal residual disease (MRD), microscopic tumor burden that persists postoperatively and remains undetectable by conventional diagnostic tools. Recent advances in liquid biopsy technologies, particularly circulating tumor DNA (ctDNA) analysis, alongside detailed characterization of the PDAC mutational landscape, offer a promising non-invasive approach for MRD detection. Emerging evidence indicates that MRD status can serve as a sensitive prognostic biomarker, identify patients at high risk of relapse, and guide personalized perioperative therapy, including optimization of adjuvant treatment. This review summarizes current knowledge on the biology and detection of MRD in PDAC, its implications for perioperative risk stratification and treatment decision-making, and discusses future directions for integrating MRD assessment into clinical practice to enable more precise, individualized patient management.

## Simple Summary

Despite curative-intent surgery, many patients with pancreatic ductal adenocarcinoma experience disease recurrence due to undetected systemic spread. Circulating tumor DNA provides a non-invasive approach to identify minimal residual disease and to detect patients at high risk of relapse earlier. Emerging evidence indicates that ctDNA has strong potential to guide personalized perioperative management and improved risk stratification. Extensive validation in well-designed prospective trials is necessary before transition to clinical implementation.

## 1. Introduction

Pancreatic ductal adenocarcinoma (PDAC) is the second most common gastrointestinal malignancy and the third leading cause of cancer-related death in the United States. According to the American Cancer Society, 67,440 new cases and 51,980 PDAC-related deaths are projected to occur in 2025 [1]. Concerningly, PDAC is estimated to overtake colorectal cancer before 2040, becoming the second leading cause of cancer-related mortality following lung cancer [2].

Although surgical resection remains the only potentially curative treatment, most patients present late during the disease course and are not considered eligible for curative-intent resection. Patients not amenable to surgery are thus treated with systemic therapies, which, despite recent advancements [3], confer little survival benefit. In the metastatic setting, NALIRIFOX (5-fluorouracil, leucovorin, liposomal irinotecan, oxaliplatin) and FOLFIRINOX (5-fluorouracil, leucovorin, irinotecan, oxaliplatin) are considered first-line combinations, achieving, however, a modest objective response rate (ORR) of 31.6% and a median overall survival (mOS) of 11.1 months [4,5].

Given the rising incidence of PDAC, clinical research efforts have focused on the optimization of treatment of patients with resectable/borderline resectable disease. Although 5-year OS rates, even after R0 resection remain around 20%, patient subgroups with prolonged survival have been identified. Cameron et al. have described a 5-year OS rate of 41% in cases with negative resection margin and no lymph node infiltration, whereas 10-year survival has been described in 11% of patients [6,7]. During the last two decades, the consolidation of interdisciplinary perioperative treatment as standard of care has greatly contributed to the improvement in outcomes of patients with radically resected PDAC. Adjuvant FOLFIRINOX offers a mOS of 54.4 months and a 3-year survival rate of 63.4% months [8]. Importantly, however, the efficacy of adjuvant chemotherapy correlates with the AJCC stage. Adjuvant chemotherapy offers no survival benefit in patients with resected subcentimeter PDAC (T1a/T1b) [9] and has limited efficacy in stage III disease [10].

The high rates of systemic relapse and distant metastasis after curative-intent surgery even for resectable PDAC suggest that a significant proportion of patients harbor minimal residual disease (MRD), microscopic tumor cells that persist after surgery and evade detection by conventional imaging, ultimately leading to recurrence. Belfiori et al. reported that approximately 10% of patients experience relapse within 12 weeks after radical resection, despite being preoperatively considered nonmetastatic based on imaging [11]. This underscores the limitations of cross-sectional imaging in detecting occult metastatic spread, resulting in disease understaging. To address these gaps, liquid biopsy has emerged as a promising noninvasive approach for MRD detection and accurate prognostication, leveraging biomarkers such as circulating tumor DNA (ctDNA) and circulating tumor cells (CTCs) to identify systemic residual disease at a molecular level. These approaches can provide much needed complementary information to enable early detection of systemic residual disease, patient risk stratification and accurate guidance of adjuvant therapeutic planning, monitoring of response and resistance to treatment and ultimately improve patient outcomes.

This review focuses on the emerging role of MRD in PDAC, highlighting its potential to refine perioperative risk stratification and guide personalized therapy. We discuss current liquid biopsy approaches for detecting subclinical disease, their prognostic value, and implications for neoadjuvant and adjuvant treatment planning. We also address future directions for integrating MRD assessment into clinical practice to improve patient outcomes.

## 2. Standard of Care Management of PDAC

### 2.1. Surgical Approach

Surgical resection remains the cornerstone of curative-intent treatment and confers the greatest level of oncological security. The principles of surgery for PDAC include removal of the tumor with an uninvolved, 1 mm tumor-free resection margin and excision of the draining lymphatic territory [12,13]. The location and extent of the tumor dictates the type of the operation. Standard resection types include pancreaticoduodenectomy (PD) for pancreas head and uncinate process tumors, distal pancreatectomy for tumors in the body-tail, total pancreatectomy for extensive or multifocal lesions and central pancreatectomy for carefully selected patients with T1 pancreatic corpus cancer [14]. Standard PD (Kausch-Whipple), pylorus-preserving PD and subtotal stomach-preserving PD are considered oncologically equivalent operations, offering comparable overall long-term survival in patients with PDAC [15,16]. Recent data has established the non-inferiority of minimally invasive pancreatectomy regarding radical resection rates, when performed in high-volume centers [17,18]. However, despite technical advancements and standardization of surgical procedures, surgery alone, although with curative intent, achieves a mOS of 17 to 20 months, necessitating systemic therapy for what appears to be a systemic disease [19,20,21].

### 2.2. Adjuvant Therapy

Adjuvant chemotherapy was not routinely used in PDAC patients until robust evidence was provided by the European Study Group for Pancreatic Cancer (ESPAC) trials in the 2000s [22]. The first randomized controlled trial comparing observation (*n* = 22 patients) to adjuvant chemoradiotherapy with fluorouracil (*n* = 21 patients) was conducted between 1974 and 1982. Although the study was prematurely terminated due to slow recruitment, patients in the experimental adjuvant arm experienced significantly prolonged mOS (20 vs. 11 months, *p* = 0.03) and disease-free survival (DFS, 11 vs. 9 months, *p* = 0.01) [23]. On the contrary, three subsequent studies failed to replicate these outcomes [24,25,26]. The ESPAC-1 trial randomized 289 patients to receive chemotherapy, chemoradiotherapy, chemoradiotherapy plus maintenance chemotherapy or observation. Patients who received chemotherapy had a significantly prolonged mOS (20.1 vs. 15.5 months) and 5-year survival rate (21% vs. 8%) compared to patients who did not receive chemotherapy. Furthermore, chemotherapy alone conferred significant survival benefit compared to observation, chemoradiotherapy or chemoradiotherapy plus chemotherapy (mOS of 21.6 vs. 16.9, 13.9 and 19.9 months, respectively) [21]. Subsequently, the CONKO-001 [19] and JSAP-02 [20] trials compared adjuvant single-agent gemcitabine versus observation and reported a consistent, statistically significant, yet clinically moderate, survival benefit with a mOS of 22.1 and 22.3 months with gemcitabine versus 20.2 and 18.4 months with observation, respectively. Gemcitabine plus adjuvant chemoradiotherapy with fluorouracil achieved a mOS of 20.5 months in the RTOG 9704 phase III study [27], consistent the findings of ESPAC-1 study reporting worse outcomes with chemoradiotherapy plus maintenance chemotherapy compared to chemotherapy alone. Similarly, in the CapRI trial, chemoradio-immunotherapy with fluorouracil, cisplatin and interferon alfa-2b plus maintenance chemotherapy did not produce superior outcomes compared to fluorouracil monotherapy, while having a worse safety profile [28]. In the late 2000s, ESPAC-3 directly compared adjuvant fluorouracil as administered in ESPAC-1 and gemcitabine as used in CONKO-001 and provided evidence for the use of gemcitabine as the preferred treatment; although mOS was similar in the two arms (23 vs. 23.6 months, respectively), gemcitabine was associated with significantly lower frequency of grade 3/4 adverse effects (7.5% vs. 14%) [29].

The results of the ESPAC-4 and PRODIGE24/CCTGPA.6 phase III trials rendered combination adjuvant chemotherapy as the modern standard of care for PDAC. ESPAC-4 randomly assigned a total of 730 patients to either single-agent gemcitabine or gemcitabine plus capecitabine. Patients in the experimental arm experienced significantly better outcomes, achieving a mOS of 28.0 months vs. 25.5 months (HR:0.82, 95%CI: 0.68–0.98, *p* = 0.032) [30]. The mOS for patients after a R0 resection was 27.9 months in the monotherapy arm and 39.5 months in the combination group. The estimated 5-year survival with gemcitabine plus capecitabine was 28.8% versus 16.3% with gemcitabine alone, significantly surpassing estimated 5-year survival rates with no treatment, gemcitabine, 5FU plus leucovorin or chemoradiotherapy, as reported in the ESPAC-1 and ESPAC-3 studies.

In the following year, the results of the phase III PRODIGE24/CCTGPA.6 (NCT01526135) were published, establishing modified-FOLFIRINOX (oxaliplatin, irinotecan, leucovorin, 5-fluorouracil) as a first-line AC combination in PDAC. A total of 493 patients were randomized to either mFOLFIRINOX or gemcitabine monotherapy. mFOLFIRINOX demonstrated significant antitumor effects, achieving a mOS of 54.4 months compared to 35.0 months in the gemcitabine arm (HR: 0.64; 95%CI: 0.48–0.86, *p* = 0.003), with a 5-year survival rate and a mDFS of 43.2% and 21.4 months versus 31.4% and 12.8 months with gemcitabine, respectively [31]. Of note, the notably longer mOS in the monotherapy group in this study was potentially due to the use of FOLFIRINOX after relapse in the gemcitabine arm.

### 2.3. Neoadjuvant Therapy

Neoadjuvant treatment (NAT) consisting of chemotherapy with or without radiotherapy, has emerged over the past decade as the center of extensive clinical research as a potential strategy to optimize outcomes, particularly in patients with borderline resectable disease who are at a higher risk for R1 resection. In patients with locally advanced PDAC, administration of neoadjuvant chemotherapy with FOLFIRINOX resulted in a successful resection in 61% of patients and significant survival benefit compared to surgical exploration alone (16 vs. 8.5 months) [32]. In addition to tumor downstaging and conversion to resectable status, additional theoretical benefits of NAC include the eradication of occult micrometastases and better patient selection based on the biologic behavior of PDAC. Intriguing genomic analyses of matched primary and metastatic PDAC revealed that the primary tumor proliferates for an average of 6.8 years before acquisition of metastatic capacity, during which progressor mutations accumulate and lead to genetically evolved metastasis-enabled subclones [33]. In another interesting study, Haeno et al. utilized a mathematical framework based on pathological and radiological data from patients with PDAC who underwent surgery and reported that all patients are expected to have metastasis-capable cells in the primary lesion, while tumors of one, two and three cm has a probability of harboring systemic metastases of 28%, 73% and 94%, respectively [34]. These predictions were subsequently validated using patient data. The authors speculated that the model predictions suggest that surgery alone does not fully eradicate the overall tumor cell burden and that earlier commencement of systemic therapy confers survival benefit by reducing the amount of cancer cells in the exponential growth stage. These data imply that PDAC behaves as a systemic disease and underline that patients with small primary tumors are also in risk of systemic tumor dissemination, favoring early initiation of systemic therapy. Additionally, up to 33% of patients do not receive adjuvant chemotherapy after PDAC resection, primarily owing to older age, worse performance status and postoperative complications [35]. Administration of NAT circumvents this hurdle, increasing the overall percentage of patients exposed to systemic therapy, which is a crucial determinant of survival.

Regarding resectable disease, the phase II SWOG S1505 clinical trial investigated perioperative (3 months prior, 3 months after) mFOLFIRINOX versus gemcitabine plus nab-paclitaxel and reported comparable outcomes between the two arms (mOS of 22.4 vs. 23.6 months and mDFS of 10.9 months vs. 14.2 months, respectively), which, importantly, were not superior to the historical standard of adjuvant-only treatment [36]. In the phase II GEMCAD 10-03 (NCT01389440) total NAC with gemcitabine plus erlotinib followed by NAC gemcitabine-erlotinib chemoradiotherapy resulted in a resectability and R0 rate of 76% and 63.1%, a mOS and a mDFS of 23.8 and 12.8 months, respectively [37]. More recently, the multicenter phase II study NORPACT-1 randomly assigned 140 patients with resectable PDAC to either NAC FOLFIRINOX or upfront surgery, followed by adjuvant chemotherapy in 86% and 90% of cases, respectively. Strikingly, NAC was associated with significantly worse outcomes with a mOS of 25.1 versus 38.5 months, while resection rates were also lower (81.8% vs. 88.9%, respectively) [38]. On the contrary, results from a phase II single-arm nonrandomized trial provided evidence of significantly improved survival and R0 resection with perioperative mFOLFIRINOX (6 cycles pre-, 6 cycles postoperatively). In the totality of the cohort (*n* = 46), the mOS and mPFS were 37.2 months and 16.6 months, while for patients that completed the study protocol (*n* = 19) mOS was not reached at the time of data cutoff (95%CI: 46.2-not reached). A total of 27 patients (59%) underwent successful surgery, of whom 93% underwent R0 resection [39].

## 3. Role of Minimal Residual Disease in Treatment Decision-Making

### 3.1. Definition

**Figure 1:**
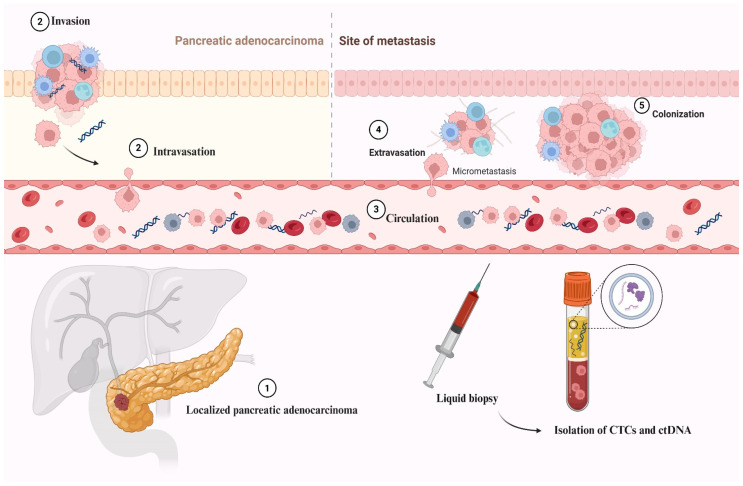
Schematic illustration of the metastatic cascade in pancreatic adenocarcinoma and the role of circulating tumor DNA (ctDNA) and circulating tumor cells (CTCs). Localized pancreatic adenocarcinoma cells (1) invade surrounding tissues and penetrate the vasculature (2, intravasation). Tumor-derived material, including CTCs and ctDNA fragments, then circulates in the bloodstream (3), serving as potential biomarkers of minimal residual disease (MRD). Circulating tumor cells may subsequently extravasate at distant sites (4), leading to micrometastatic deposits and, eventually, metastatic colonization (5). Liquid biopsy enables noninvasive detection and molecular profiling of CTCs and ctDNA, providing real-time insights into tumor dynamics, recurrence risk, and treatment response.

Minimal residual disease refers to the minute number of residual viable cancer cells that persist after curative-intent treatment and are undetectable by conventional imaging methods. Detecting MRD is crucial for assessing treatment efficacy, predicting relapse and informing decisions on adjuvant therapy. The clinical utility of MRD detection is well established in hematological malignancies and is gradually gaining momentum in solid tumors [40,41]. Several biomarkers have been developed to detect MRD with high sensitivity, the most prominent being circulating tumor DNA (ctDNA) and circulating tumor cells (CTCs). CTCs comprise intact tumor cells that have detached from the primary or metastatic site and entered the circulation, while ctDNA reflects tumor-specific genetic material shed into the bloodstream (Figure 1). Together, these liquid biopsy tools provide a non-invasive, real-time dynamic window into the presence of residual disease and the risk of recurrence. Over the past few years, detailed characterization of the PDAC genomic profile and significant technological advances have enabled the detection of very small numbers of CTCs and minute amounts of ctDNA [42,43].

#### 3.1.1. ctDNA

ctDNA refers to the fraction of cell-free DNA that originates from cancer cells, primarily upon cell death via apoptosis and necrosis or via active secretion through extracellular vesicles. In cases of complete resection of the primary tumor, ctDNA is derived from occult micrometastases or residual disseminated tumor cells. There exist two main strategies for ctDNA detection, targeted, tumor-informed tests and untargeted, tumor agnostic tests [44]. Tumor-informed assays rely on initial sequencing of patients’ tumor tissues to identify tumor-specific mutations, enabling the design of customized probes for highly sensitive ctDNA detection. In contrast, uninformed tests involve untargeted screening of point mutations by whole-genome sequencing or whole exome sequencing. This approach harbors the risk of false-positive findings arising from detecting background genetic aberrations of non-malignant origin [45]. Polymerase chain reaction (PCR) is most effective in tumor-informed testing, where known mutations can be precisely targeted using digital or quantitative PCR, whereas it is of limited utility in a tumor-agnostic approach because it cannot broadly screen for unknown mutations. Next-generation sequencing (NGS) is used in both tumor-agnostic and tumor-informed settings; In a tumor-agnostic approach, NGS enables comprehensive genomic profiling to identify mutations without prior knowledge. In a tumor-informed approach, NGS can be used to detect sequence-specific regions harboring clinically significant mutations, followed by amplification of the target region [46]. In PDAC, Watanabe et al. demonstrated superior detection rate with the tumor-informed compared to the tumor-agnostic approach in the immediate postoperative period both for treatment-naïve patients (56% vs. 39%) and neoadjuvant-treated patients (36% vs. 31%, respectively) [47].

#### 3.1.2. Circulating Tumor Cells

CTCs are malignant cells that detach from the primary tumor or metastatic foci and disseminate into the bloodstream, acting as key mediators of the metastatic cascade. To acquire invasive and migratory properties, tumor cells undergo epithelial-to-mesenchymal transition (EMT), a process that enhances motility and confers resistance to apoptosis [48]. Upon reaching distant organs, these cells often revert to an epithelial phenotype through mesenchymal-to-epithelial transition (MET), which supports their adhesion, proliferation, and the development of secondary lesions [49]. A major challenge in CTC detection is their scarcity and phenotypic heterogeneity. The epithelial cell adhesion molecule (EpCAM) remains one of the most frequently utilized targets for CTC identification [50]. Popular CTC detection assays, including CellSearch and AdnaTest CTC Select, use immunomagnetic isolation strategies that depend on EpCAM expression. However, CTCs that have undergone EMT often downregulate epithelial markers, rendering EpCAM-based platforms less sensitive and likely to underestimate their actual frequency [51]. Importantly, however, in PDAC, both the overall CTC burden and the proportion of cells exhibiting mesenchymal characteristics increase with tumor stage, aiding in the differentiation of localized from metastatic disease [52]. Besides EpCAM, common epithelial markers include cytokeratins 8, 18, and 19, whereas mesenchymal phenotypes are often characterized by the expression of vimentin, Twist, and KLF8. Beyond immunoaffinity-based capture, CTCs can also be isolated through physical properties. Label-free approaches exploit differences in density, size, deformability, or electric charge, as in density gradient centrifugation, filtration systems, functionalized micro-surfaces, or microfluidic devices [53].

### 3.2. Prognostic Significance of MRD

To date, several studies have investigated the prognostic significance of MRD detection after curative-intent resection for PDAC (Table 1). In the post-operative setting ctDNA positivity has been reported in 19.3% to 51.8%, in patients with locally advanced disease after NAC and resection [54,55,56,57], indicating a significant percentage of patients with molecular residual disease. In resected PDAC, ctDNA positivity precedes postoperative radiographic recurrence in more than 50% of cases with a median lead time of 5.5 [58] and 6.5 months [59]. According to a recent meta-analysis of nine studies, postoperatively detectable *KRAS*-mutated ctDNA was associated with worse mRFS (HR:3.32; 95%CI: 2.19–5.03, *p* < 0.001) and worse mOS (HR: 6.62; 95% CI: 2.18–20.16, *p* < 0.001) compared to negative ctDNA [60]. A strikingly shorter RFS was observed in patients with detectable ctDNA pre- and post-operatively versus consistently ctDNA-negative patients (4.7 vs. 22.3 months, *p* < 0.001) [61]. Non-touch isolation techniques [62] and portal vein sampling [63] had no impact on liquid biopsy status. In patients who underwent NAC, positive postoperative ctDNA had no effect on mRFS, while it was inversely correlated with mOS (*p* = 0.014) [57].

An inverse relationship between postoperative MRD, RFS and OS was also observed in several subsequent studies. In the largest cohort analysis to date, positive ctDNA within three months from the operation was correlated with significantly shorter median disease-free survival (DFS of 6.3 vs. 33.3 months, HR: 4.45; 95%CI: 2.94–10.1, *p* < 0.0001), which was consistently observed across all AJCC stages. Additionally, patients with *KRAS*-wildtype ctDNA had significantly longer DFS compared to *KRAS*-mutant ctDNA (*p* = 0.02) [55]. In the study of Dickey et al., ctDNA positivity was observed in 28.1% of patients, including 14.3% of patients with upfront resectable disease and 54.5% of patients with borderline resectable tumors [56]. Preceding or simultaneous ctDNA positive test was reported in 47.4% of patients with eventual radiographic recurrence. ctDNA+ patients had significantly lower mRFS (3.6 vs. 29 months, *p* < 0.001) and mOS (26.3 months vs. not reached, *p* < 0.001) compared to ctDNA- patients. In another study by Li et al., detectable ctDNA in the MRD window was associated with significantly shorter mRFS (6.6 vs. 25.0 months) and mOS (25.5 vs. not reached) [58]. Lastly, according to data from a nonrandomized clinical trial testing perioperative mFOLFIRINOX in resectable disease, baseline ctDNA was not associated with survival, while postoperative ctDNA-negative status was significantly associated with PFS (HR: 34.0; 95%CI: 2.6–4758.6, *p* = 0.006 and OS (HR: 11.7; 95%CI: 1.5–129.9, *p* = 0.02) [39].

Peritoneal cell-free tumor DNA (ptDNA) is a much less studied index of MRD. In PDAC, the peritoneum is the second most common metastatic site and peritoneal carcinomatosis is present in approximately 9% of patients at the time of diagnosis and up to 50% at the time of death [64]. Leick et al. investigated the utility of postresection peritoneal cfDNA in predicting locoregional and peritoneal relapse [65]. Postresection peritoneal lavage fluid was collected intraoperatively after curative-intent pancreatectomy. *KRAS*-mutant ptDNA was detected in 52% of preresection and 83% postresection samples, likely reflecting the effect of tumor manipulation. A mutant allele frequency >0.1% was correlated with significantly shorter locoregional and peritoneal, but not distant, mRFS compared to a MAF of <0.1% (8.9 months vs. not reached, *p* = 0.003).

**Table 1:** Summary of studies on the prognostic role of postoperative ctDNA detection. Abbreviations: mOS: median overall survival; mRFS: median recurrence free survival; mDFS: median disease-free survival; HR: hazard ratio; CI: confidence intervals; NR: not reached; N/A not available.

Study, Year	Type of Study	Patients	ctDNA Positivity, %	mOS		mRFS		mDFS		Reference
				Months	HR	Months	HR	Months	HR	
Pietrasz et al., 2017	Prospective, single-center	36	16.6%	19.3 vs. 32.2	N/A	N/A	N/A	4.6 vs. 17.6	N/A	[54]
Nakano et al., 2018	Retrospective, single-center	45	44.4%	N/A	Univariate 3.18, 95%CI: 0.95–10.65	N/A	N/A	N/A	Univariate: 2.2, 95%CI: 0.99–4.87 Multivariate: 2.91, 95%CI: 1.10–5.61	[66]
Botta et al., 2024	Retrospective, multi-center	100	29%	N/A	N/A	N/A	N/A	6.3 vs. 33.3	5.4, 95%CI: 2.9–10.1	[55]
Lee et al., 2019	Prospective, multi-center	35	37%	N/A	4.0, 95%CI: 1.2–13.6	N/A	Univariate: 5.4, 95%: CI 1.9–25.2 Multivariate: 6.3; 95% CI:2.4–16.2	N/A	N/A	[67]
Dickey et al., 2025	Retrospective, single-center	32	28.1%	26.3 vs. NR	N/A	3.6 vs. 29.0	72.1, 95%CI: 8.6–604.9	N/A	N/A	[56]
Li et al., 2025	Retrospective, two-institution	27	N/A	25.5 vs. NR	2.5, 95%CI: 0.6–10.2	6.6 vs. 25.0	3.1, 95%CI: 1.0–9.4	N/A	N/A	[58]
Cecchini et al., 2024	phase 2 nonrandomized controlled trial	12	17%	N/A	Univariate: 11.7; 95%CI: 1.5–129.9	N/A	34.0; 95%CI: 2.6–4758.6	N/A	N/A	[39]
Kitahata et al., 2022	Prospective, single-center	27	51.8%	23.8 vs. NR	5.01, 95%CI: 1.22–20.51	11.1 vs. 12.3	N/A	N/A	N/A	[57]
Groot et al., 2019	Prospective, single-center	41	26.8%	N/A	N/A	5.0 vs. 15.0	N/A	N/A	N/A	[68]
Yamaguchi et al., 2021	Retrospective, single-center	97	28.0%	Pre+/post+: 13.5 Pre−/post+: 22.2 Pre−/post−: 52.6 Pre+/post−: 18.2	Multivariate: 1.35; 95%CI: 0.75–2.39	Pre+/post+: 4.7 Pre−/post+: 13.1 Pre−/post−: 22.3 Pre+/post−: 10.9	Multivariate: 1.61, 95%CI: 0.90–2.77	N/A	N/A	[61]
Hata et al., 2023	Retrospective, single-center	66	24.2%	N/A	Univariate: 2.7, 95%CI: 1.1–6.7 Multivariate: 2.1, 95%CI: 0.8–5.4	N/A	N/A	N/A	Univariate: 2.1, 95%CI: 1.03–4.3 Multivariate: 2.7, 95%CI: 1.3–5.7	[69]

### 3.3. MRD in Guiding Adjuvant Strategies

The potential for post-operative risk stratification to guide adjuvant chemotherapy allocation based on liquid biopsy status has been explored in several studies, particularly in patients with colorectal cancer. The DYNAMIC study randomly assigned 455 patients with stage II CRC to receive adjuvant treatment based on either detectable postoperative ctDNA levels or standard high-risk clinicopathological features. ctDNA-guided management was not inferior to standard decision-making in terms of 5-year RFS (88% vs. 87%, respectively) [70], while it resulted to significantly reduced percentage of treated patients (15% vs. 28%, RR:1.82; 95%CI: 1.25–2.65) [71]. Several trials investigating MRD-guided adjuvant management in CRC are also currently ongoing, exploring either administration of intensified regiments to ctDNA+ or de-escalation in ctDNA-negative patients [72].

Regarding PDAC, the multicenter AGITG DYNAMIC-Pancreas trial evaluated the role of ctDNA testing after upfront surgery in guiding adjuvant chemotherapy for early stage PDAC. According to the study protocol, ctDNA+ patients were scheduled to receive 6 months of AC, while ctDNA- patients could de-escalate to 3 months of treatment based on clinician’s discretion. A total of 102 patients were recruited, of which 53% were ctDNA-; Among ctDNA- patients, 44% were administered 3 months of AC. After a median follow-up of 36 months, ctDNA- patients experienced significantly prolonged mRFS compared to ctDNA+ patients (22 vs. 13 months, *p* = 0.003), indicating the feasibility of MRD-guided decision-making in the adjuvant setting [73].

Furthermore, Botta et al. reported that patients who received NAC and had positive ctDNA in the immediate postoperative period did not experience a survival benefit with the addition of adjuvant chemotherapy [55]. This finding potentially suggests chemoresistant disease in this patient subgroup who should be, therefore, offered an adjuvant regiment different from NAC or be treated in a clinical trial setting. Unexpectedly, patients with negative tumor-informed ctDNA after NEC who received adjuvant treatment had shorted mDFS compared to patients under surveillance. This finding should be interpreted with caution and could be potentially explained by the presence of high-risk pathologic features in patients who received adjuvant chemotherapy.

### 3.4. Limitations of Current Studies

Despite the growing body of literature, the available studies are characterized by certain methodological limitations that must be acknowledged to appropriately contextualize their findings. Most published studies to date are retrospective and/or single-center in nature, with relative small cohorts (mean sample size of approximately 47 patients, Table 1), limiting generalizability and increasing susceptibility to bias and confounding. Additionally, the majority of studies used assays targeting *KRAS* mutations, which may result in false-negative ctDNA findings in patients with *KRAS* wild-type tumors, potentially underestimating the residual tumor burden. This phenomenon is clinically relevant as *KRAS* mutations are detected in 81% of PDAC patients, meaning that approximately 20% of patients may have false negative results [43]. Preoperative ctDNA measurements were not uniformly available, limiting interpretation of postoperative ctDNA dynamics and assessment of baseline ctDNA shedding. Lastly, substantial variability in the timing and frequency of postoperative blood sampling was observed, ranging from as early as 3 days after surgery to as late as 8 weeks, with some studies relying on single time-point assessments and others incorporating longitudinal sampling.

Although disease-specific, PDAC-tailored ctDNA practice guidelines are not yet established, the European Society for Medical Oncology (ESMO) has issued cross-tumor recommendations that frame standardized ctDNA sampling time-points after curative-intent surgery: postoperatively to define MRD status and inform escalation/de-escalation of adjuvant strategies, after completion of standard adjuvant therapy to identify the need for second-line therapy, longitudinally during surveillance to evaluate whether ctDNA can detect relapse earlier than conventional follow-up and whether ctDNA-informed surveillance algorithms improve outcomes [74].

### 3.5. Limitations and Challenges in MRD Detection

Despite notable technological advancements in ctDNA analysis, the preoperative and postoperative detection rate in localized solid tumors remains limited, primarily reflecting intrinsic tumor biological characteristics [68]. This observation is clinically pertinent, as it is reflected in a subset of patients with negative postoperative ctDNA who subsequently develop metastatic disease.

Low ctDNA shedding, a desmoplastic microenvironment, poor vascularization or anatomical barriers constitute potential factors that hinder ctDNA detection. PDAC tumor microenvironment is characterized by high stromal and low cellular features. To this end, the fraction of ctDNA-positive patients is lower in localized PDAC compared to gastroesophageal and colorectal cancer [75]. Furthermore, the rate of ctDNA detection appears to be influenced by the micrometastatic site. In resected colorectal cancer, lower median ctDNA levels were observed in cases of lung or peritoneal metastases and higher in liver metastases [76]. Similarly, in PDAC metastatic site was the most important determinant of ctDNA levels, with liver metastases being associated with significantly higher ctDNA levels compared to extrahepatic metastatic sites [77]. Regarding studies of perioperative chemotherapy, personalized tumor-informed testing may not be feasible in patients with major pathologic responses who have insufficient tissue for sequencing. Although, baseline tissue specimens could, theoretically, be used for this purpose, NAC significantly impacts the somatic mutation status [78], rendering the reliability of informed ctDNA testing questionable. In such cases, tumor-agnostic MRD detection would be the most reliable alternative.

Furthermore, study design may structurally predispose to false-negative ctDNA results, as most published studies rely on assays targeting *KRAS* hotspot alterations, which may fail to detect MRD in patients with *KRAS*–wild-type tumors. Even in tumor-informed approaches using next-generation sequencing of the primary tumor, clinically relevant mutations may be missed because of intratumoral heterogeneity [79] or spatial sampling bias, resulting in incomplete representation of the genomic landscape of residual disease.

These assay-related limitations are particularly relevant when ctDNA is used to inform adjuvant treatment decisions, where false reassurance from ctDNA negativity could lead to inappropriate treatment de-escalation. Accordingly, although preliminary data support the technical feasibility and prognostic potential of postoperative ctDNA assessment, robust conclusions regarding its clinical utility require large-scale, prospective randomized studies to establish non-inferiority, define appropriate clinical thresholds, and confirm safety before MRD-guided strategies can be adopted in routine surgical oncology practice.

## 4. Future Perspectives

### 4.1. Personalizing Perioperative Treatment

Current evidence suggests a significant potential for ctDNA analysis in guiding perioperative treatment decisions. In the preoperative setting, baseline ctDNA is associated with worse outcomes [80] and could, in principle, identify high-risk patients, complement anatomy-based staging and guide clinical decision-making for upfront surgery versus NAC [81]. Post-NAC, ctDNA presence was associated with worse outcomes [80], while ctDNA clearance was associated with improved overall survival [82]. These observations provide a biologic rationale for using post-NAC ctDNA positivity as a marker of aggressive or systemic disease, potentially prompting intensified perioperative strategies. To this end, active clinical trials are evaluating ctDNA for NAC response assessment and prediction of resectability in borderline resectable cases [83].

Postoperative ctDNA status can provide insights into patient prognosis and provide answers to three clinical quarries: (1) Does the patient respond to the given perioperative regimen? (2) Does the patient need treatment intensification? (3) Does the patient need AC de-escalation? Given that a small percentage of patients do progress under NAC, change in ctDNA status from negative to positive or persistence of ctDNA positivity after NAC and surgical resection may indicate lack of response, chemoresistance, aggressive tumor biology and the need for switching to another chemotherapeutic regimen. To this end, patients with undetectable preoperative but detectable postoperative ctDNA had shorter mRFS (13.1 vs. 22.3 months) and mOS (22.2 vs. 52.6 months) compared to persistently ctDNA-negative patients [61]. Detectable ctDNA both pre- and postoperatively was associated with strikingly worse outcomes compared to pre-negative/post-negative and even pre-negative/post-positive status [61]. Switch-therapy or treatment intensification versus treatment continuation for persistent positive or converted positive ctDNA patients would worth investigation in clinical trial settings, given that these patients are in very high-risk for recurrence.

Consideration of treatment de-escalation requires highly accurate predictive biomarkers to minimize the chance of undertreating patients. Based on data from the ESPAC-3 study, completion of the full 6 cycles of AC was significantly associated with better outcomes compared to less than 6 cycles (HR: 0.516; 95%CI: 0.44–0.60, *p* < 0.001) [84]. Interestingly, however, preliminary data from the AGITG DYNAMIC-Pancreas trial proved the feasibility of ctDNA in guiding adjuvant treatment de-escalation. An intriguing concern to be addressed, however, is whether ctDNA-negative patients who will progress after de-escalated treatment completion will be adequately salvaged following treatment resumption. Routine adoption of ctDNA-guided de-escalation in the adjuvant setting will necessitate non-inferiority confirmation from high-level randomized trials with well-defined, uniform, central sample collection schedules and analysis.

Contrary to ctDNA, ptDNA testing is considerably less studied and, thus, insights into the management of ptDNA patients are lacking. Leick et al. reported an association of ptDNA with locoregional and peritoneal recurrence, but not distant metastases, indicating that data on the prognostic significance of ctDNA should be only carefully extrapolated in ptDNA-positive patients [65]. Further studies are required to investigate ptDNA and its impact on patient survival and adjuvant systemic chemotherapy planning. Intraperitoneal chemotherapy has been used for peritoneal metastases from intestinal and gynecological cancers, however, less data exist on PDAC [85]. Sugarbaker et al. evaluated adjuvant prophylactic hyperthermic intraperitoneal chemotherapy (HIPEC) with gemcitabine plus 6 months of normothermic intraperitoneal chemotherapy with gemcitabine in 8 patients with resected PDAC [86]. The authors reported a mOS of 29 months, which is higher compared to 23.6 with adjuvant intravenous single agent gemcitabine in the ESPAC-3 trial. In another study, 39 patients were treated with adjuvant HIPEC with (46.2%) or without adjuvant systemic chemotherapy. The mOS was 17 months and the 5-year OS rate was 24%, while adjuvant chemotherapy was not significantly associated with outcomes [87]. When compared to the outcomes of single-agent adjuvant chemotherapy, which was first-line at the time of patient recruitment in these studies, adjuvant intraperitoneal chemotherapy appears to confer a survival benefit. Selection of patients based on ptDNA status and personalized administration of intraperitoneal chemotherapy coupled with multi-agent systemic chemotherapy could be investigated in a clinical trial setting.

### 4.2. Ongoing Clinical Trials and Research Directions

Several ongoing clinical trials are investigating the prognostic significance of MRD and the utility of ctDNA as an index of MRD (Table 2). FRENCH.MRD.PDAC (NCT06287749) is an observational study aiming to confirm that ctDNA, detected after curative intent surgery and after completion of adjuvant treatment, is a marker of MRD and correlates with DFS. Additionally, this study will investigate the effect of adjuvant chemotherapy on ctDNA levels and the time difference between molecular and clinical recurrence, as secondary objectives. Similarly, GUIDEMRD (NCT06102889) aims to determine the association between ctDNA status after curative intent surgery and after adjuvant chemotherapy with mDFS in PDAC patients. The CIRCPAC trial (NCT05788744) is a currently recruiting study evaluating the prognostic value of perioperative ctDNA levels in predicting recurrence and overall survival and whether ctDNA-guided surveillance may improve outcomes compared to standard postoperative follow-up. ORACLE (NCT05059444) is another observational study investigating the utility of ctDNA testing, as surrogate for MRD, for clinical recurrence prediction after treatment of early-stage solid tumors, including PDAC.

Furthermore, NCT06867146 is designed to assess the role of MRD in guiding the decision-making regarding adjuvant treatment in patients with resected PDAC. The primary endpoints the association of peripheral-blood MRD with mDFS under multi-agent AC, aiming to identify patients who derive the greatest benefit from adjuvant therapy. The TESLA trial, an active, randomized, interventional phase II study (NCT05638698) is investigating maintenance therapy with TG01/QS-21 vaccine with or without balstilimab in patients who remain MRD+ after completion of standard adjuvant chemotherapy for stage I-III *KRAS*-mutant PDAC [88]. The trial’s primary objective is the 6-month molecular disease control rate, defined as stable, decreased or cleared ctDNA. ADAPT-MRD (NCT06966440) is a recruiting, randomized, interventional study investigating the impact of ct-DNA-guided adjuvant treatment on DFS. According to study protocol, patients are randomized 1:1 to either standard of care adjuvant chemotherapy or adjuvant chemotherapy with serial MRD assessment; those achieving two consecutive negative MRD tests undergo treatment de-escalation, whereas persistent MRD positivity prompts therapy escalation beyond the standard 6-month course.

### 4.3. ctDNA Within Multimarker Risk Stratification Frameworks

Apart from stand-alone marker, ctDNA could be used within integrated multimarker risk profiles in an attempt to mitigate limitations inherent to ctDNA assays and individual biomarkers. To this end, using data from the PANACHE01-PRODIGE48 trial, Pinson et al. showed that patients who were simultaneously CA19-9 high and ctDNA-positive had significantly lower survival than patients that were not [89]. Future strategies may extend this approach by combining ctDNA with tissue-based personalized biomarkers that are associated with tumor biology and treatment sensitivity. Several immunohistochemistry-based markers have shown reproducible associations with outcomes and therapy response. GATA6 expression distinguishes classical from basal-like tumor subtypes and has been linked to prognosis and chemotherapy response [90]. Similarly, Keratin 17 has been associated with inferior survival [39], while the lack of expression of class III β-tubulin [91] and the expression of human equilibrative nucleoside transporter 1 (hENT1) have been correlated with chemotherapy response and longer survival [92].

### 4.4. Translational Research Opportunities

The rapid advent of artificial intelligence (AI) in medicine has revolutionized various fields, from diagnostic imaging and drug discovery to personalized treatment strategies and predictive analytics [93,94,95]. By harnessing large amounts of data, AI holds promise for refining and improving MRD detection and MRD-guided decision-making by enhancing sensitivity, automating data analysis and integrating predictive modeling. A major limitation of incorporating routine ctDNA analysis into perioperative decision-making is that not all tumors release sufficient ctDNA into systemic circulation, especially in earlier stages, leading to false-negative results and impacting cost-effectiveness [75,96]. ML models could address this barrier by prognosticating which patients are most likely to benefit from it. By analyzing large datasets that integrate tumor characteristics, patient demographics, genomic characteristics and prior ctDNA shedding patterns, Ml models could identify key predictors of ctDNA status. To this end, Shin et al. reported a logistic regression ML model that effectively predicted lung cancer patients with detectable ctDNA based on clinical factors (AUC of 0.77, accuracy of 71.8%) [97]. Furthermore, ML could also play a role in overcoming the ctDNA signal sparsity in low TF conditions and increase MRD detection sensitivity. Widman et al. introduced MRD-EDGE, an AI-guided ctDNA analysis platform, which integrates complementary signals from single nucleotide variants and copy number variants to achieve ultra-sensitive ctDNA detection [98]. More specifically, MRD-EDGE uses a deep learning classifier that distinguishes true ctDNA mutations from the far more abundant sequencing errors, decreasing noise and enriching signal. Furthermore, for CNVs, MRD-EDGE employs ML-based read-depth denoising along with an expanded feature set that incorporates fragmentomics and allelic frequency analysis of germline single nucleotide polymorphisms, thereby enabling ctDNA detection even at low aneuploidy levels. This platform demonstrated excellent performance for ctDNA SNV detection in early-stage CRC patients (AUC of 1.00, sensitivity of 100% and specificity of 90%) and accurately tracked response to neoadjuvant chemotherapy in lung cancer and immunotherapy in metastatic melanoma. Zhu et al. developed a DL model (Fragle) for the accurate quantification of ctDNA based on cfDNA fragment length density distribution, with no requirement for tumor biopsy. Further studies have reported effective DL models for the automatic detection, classification and viability assessment of CTCs [99,100,101].

## 5. Conclusions

Despite decades of surgical and systemic advances, PDAC remains a major clinical challenge, with recurrence after curative-intent resection continuing to dictate patient prognosis. The emerging evidence on MRD exposes a concerning reality: for a subset of patients, surgical resection may be futile, as preoperative occult systemic disease persists undetected. Liquid biopsy provides provocative insights into the invisible biology of PDAC. Patients with persistent or newly detectable ctDNA represent a high-risk population whose disease is not yet visible, but it will eventually lead to clinical recurrence.

The potential of MRD monitoring extends beyond prognosis. It raises urgent questions about the timing, selection, and intensity of systemic therapy: when is early intervention truly transformative and when does biology simply outpace our tools? Should we reconsider current adjuvant regimens and explore molecularly guided escalation or novel interventions in these patients? Conversely, in ctDNA-negative patients, could carefully tailored de-escalation reduce toxicity without compromising outcomes? Could integration of AI and machine learning into MRD detection enable us to anticipate relapse rather than respond to it, transforming perioperative care from reactive to preemptive?

At the same time, it must be emphasized that, despite promising early findings, the current evidence remains insufficient to support routine use of postoperative ctDNA as a stand-alone tool for guiding clinical management. Definitive translation into clinical practice will require adequately powered, prospective, randomized, multicenter studies with standardized assays and sampling intervals in order to validate prognostication performance and assess the safety and efficacy of ctDNA-guided treatment adaptation. Only upon such extensive validation can MRD-directed strategies be advanced from an investigational biomarker to a reliable component of contemporary surgical oncology practice.

Ultimately, these data challenge the field to reconsider traditional definitions of resectability and cure in PDAC. Surgical success should not be defined by what is removed from the patient, but by what remains unseen, leading to biologic R2 resections. Future management of PDAC should embrace a biology-driven, precision approach that anticipates recurrence before it manifests, fundamentally altering the natural history of PDAC.

## Figures and Tables

**Table 2:** Overview of ongoing studies evaluating postoperative ctDNA as a prognostic biomarker and as a tool to guide adjuvant therapy decisions.

National 06287749.	Study Type	Status	Primary Endpoint
NCT06287749 (FRENCH.MRD.PDAC)	Observational	Recruiting	Association between DFS and ctDNA status after curative-intent surgery and adjuvant chemotherapy
NCT06102889 (GUIDEMRD)	Observational	Enrolling by invitation	Association between DFS and ctDNA status after curative-intent surgery and adjuvant chemotherapy
NCT05788744 (CIRCPAC)	Interventional	Recruiting	(1) Association between preoperative ctDNA status and early recurrence (2) Association between preoperative eccDNA status and early recurrence (3) Association between ctDNA status and DFS (4) OS of patients with preoperative ctDNA detection vs. preoperative eccDNA detection (5) Association of DFS with eccDNA status
NCT05059444 (ORACLE)	Observational	Recruiting	Association between distant recurrence free interval and postoperative ctDNA status
NCT06867146	Interventional	Active, not recruiting	Association of DFS with MRD
NCT05638698 (TESLA)	Interventional	Active, not recruiting	6-month molecular disease control rate, as defined by ctDNA stability, decrease or clearance in patient with ctDNA+ resected PDAC with no evidence of disease on imaging, treated with TG01, QS-21 or Balstilimab
NCT06966440 (ADAPT-MRD)	Interventional	Active	Impact of ct-DNA-guided adjuvant therapy on DFS

## Data Availability

Data supporting the recommendations of this article are included within the reference list. Please contact the corresponding author for any further data request.

## References

[1] Siegel R.L., Kratzer T.B., Giaquinto A.N., Sung H., Jemal A. (2025). Cancer statistics, 2025. CA Cancer J Clin.

[2] Cronin K.A., Scott S., Firth A.U., Sung H., Henley S.J., Sherman R.L., Siegel R.L., Anderson R.N., Kohler B.A., Benard V.B. (2022). Annual report to the nation on the status of cancer, part 1: National cancer statistics. Cancer.

[3] Theocharopoulos C., Ziogas I.A., Douligeris C.-C., Efstathiou A., Kolorizos E., Ziogas D.C., Kontis E. (2024). Antibody-drug conjugates for hepato-pancreato-biliary malignancies: “Magic bullets” to the rescue?. Cancer Treat. Rev..

[4] Conroy T., Desseigne F., Ychou M., Bouche O., Guimbaud R., Becouarn Y., Adenis A., Raoul J.L., Gourgou-Bourgade S., de la Fouchardiere C. (2011). FOLFIRINOX versus gemcitabine for metastatic pancreatic cancer. N. Engl. J. Med..

[5] Wainberg Z.A., Melisi D., Macarulla T., Pazo Cid R., Chandana S.R., De La Fouchardiere C., Dean A., Kiss I., Lee W.J., Goetze T.O. (2023). NALIRIFOX versus nab-paclitaxel and gemcitabine in treatment-naive patients with metastatic pancreatic ductal adenocarcinoma (NAPOLI 3): A randomised, open-label, phase 3 trial. Lancet.

[6] Winter J.M., Cameron J.L., Campbell K.A., Arnold M.A., Chang D.C., Coleman J., Hodgin M.B., Sauter P.K., Hruban R.H., Riall T.S. (2006). 1423 pancreaticoduodenectomies for pancreatic cancer: A single-institution experience. J. Gastrointest. Surg..

[7] Cameron J.L., Riall T.S., Coleman J., Belcher K.A. (2006). One thousand consecutive pancreaticoduodenectomies. Ann. Surg..

[8] Conroy T., Hammel P., Hebbar M., Ben Abdelghani M., Wei A.C., Raoul J.L., Chone L., Francois E., Artru P., Biagi J.J. (2018). FOLFIRINOX or Gemcitabine as Adjuvant Therapy for Pancreatic Cancer. N. Engl. J. Med..

[9] Shaib W.L., Narayan A.S., Switchenko J.M., Kane S.R., Wu C., Akce M., Alese O.B., Patel P.R., Maithel S.K., Sarmiento J.M. (2019). Role of adjuvant therapy in resected stage IA subcentimeter (T1a/T1b) pancreatic cancer. Cancer.

[10] Li X.H., Zhou E.L., Dong X.Y., Zhao C.Y., Han Y.X., Cui B.K., Lin X.J. (2023). Adjuvant chemotherapy in pancreatic cancer at different AJCC stages: A propensity score matching analysis. Eur. J. Med. Res..

[11] Belfiori G., Crippa S., Pagnanelli M., Gasparini G., Aleotti F., Camisa P.R., Partelli S., Pecorelli N., De Stefano F., Schiavo Lena M. (2024). Very Early Recurrence After Curative Resection for Pancreatic Ductal Adenocarcinoma: Proof of Concept for a “Biological R2 Definition”. Ann. Surg. Oncol..

[12] Tol J.A., Gouma D.J., Bassi C., Dervenis C., Montorsi M., Adham M., Andren-Sandberg A., Asbun H.J., Bockhorn M., Buchler M.W. (2014). Definition of a standard lymphadenectomy in surgery for pancreatic ductal adenocarcinoma: A consensus statement by the International Study Group on Pancreatic Surgery (ISGPS). Surgery.

[13] Leonhardt C.S., Niesen W., Kalkum E., Klotz R., Hank T., Buchler M.W., Strobel O., Probst P. (2022). Prognostic relevance of the revised R status definition in pancreatic cancer: Meta-analysis. BJS Open.

[14] Terai T., Kawai M., Kitahata Y., Satoi S., Hashimoto D., Nagai M., Nishiwada S., Yamamoto T., Yamaue H., Sho M. (2023). Central pancreatectomy might be an acceptable surgical procedure for clinical T1 pancreatic body ductal adenocarcinoma: A multicenter retrospective analysis. J. Hepato-Biliary-Pancreat. Sci..

[15] Calderon E., Day R.W., Stucky C.C., Gray R.J., Pockaj B.A., Chang Y.H., Wasif N. (2020). Comparative Effectiveness of Pylorus-Preserving Versus Standard Pancreaticoduodenectomy in Clinical Practice. Pancreas.

[16] Tran K.T., Smeenk H.G., van Eijck C.H., Kazemier G., Hop W.C., Greve J.W., Terpstra O.T., Zijlstra J.A., Klinkert P., Jeekel H. (2004). Pylorus preserving pancreaticoduodenectomy versus standard Whipple procedure: A prospective, randomized, multicenter analysis of 170 patients with pancreatic and periampullary tumors. Ann. Surg..

[17] Klotz R., Mihaljevic A.L., Kulu Y., Sander A., Klose C., Behnisch R., Joos M.C., Kalkum E., Nickel F., Knebel P. (2024). Robotic versus open partial pancreatoduodenectomy (EUROPA): A randomised controlled stage 2b trial. Lancet Reg. Health Eur..

[18] Korrel M., Jones L.R., van Hilst J., Balzano G., Bjornsson B., Boggi U., Bratlie S.O., Busch O.R., Butturini G., Capretti G. (2023). Minimally invasive versus open distal pancreatectomy for resectable pancreatic cancer (DIPLOMA): An international randomised non-inferiority trial. Lancet Reg. Health Eur..

[19] Oettle H., Post S., Neuhaus P., Gellert K., Langrehr J., Ridwelski K., Schramm H., Fahlke J., Zuelke C., Burkart C. (2007). Adjuvant chemotherapy with gemcitabine vs. observation in patients undergoing curative-intent resection of pancreatic cancer: A randomized controlled trial. JAMA.

[20] Ueno H., Kosuge T., Matsuyama Y., Yamamoto J., Nakao A., Egawa S., Doi R., Monden M., Hatori T., Tanaka M. (2009). A randomised phase III trial comparing gemcitabine with surgery-only in patients with resected pancreatic cancer: Japanese Study Group of Adjuvant Therapy for Pancreatic Cancer. Br. J. Cancer.

[21] Neoptolemos J.P., Stocken D.D., Friess H., Bassi C., Dunn J.A., Hickey H., Beger H., Fernandez-Cruz L., Dervenis C., Lacaine F. (2004). A randomized trial of chemoradiotherapy and chemotherapy after resection of pancreatic cancer. N. Engl. J. Med..

[22] Klaiber U., Hackert T., Neoptolemos J.P. (2019). Adjuvant treatment for pancreatic cancer. Transl. Gastroenterol. Hepatol..

[23] Kalser M.H., Ellenberg S.S. (1985). Pancreatic cancer. Adjuvant combined radiation and chemotherapy following curative resection. Arch. Surg..

[24] Bakkevold K.E., Arnesjo B., Dahl O., Kambestad B. (1993). Adjuvant combination chemotherapy (AMF) following radical resection of carcinoma of the pancreas and papilla of Vater--results of a controlled, prospective, randomised multicentre study. Eur. J. Cancer.

[25] Klinkenbijl J.H., Jeekel J., Sahmoud T., van Pel R., Couvreur M.L., Veenhof C.H., Arnaud J.P., Gonzalez D.G., de Wit L.T., Hennipman A. (1999). Adjuvant radiotherapy and 5-fluorouracil after curative resection of cancer of the pancreas and periampullary region: Phase III trial of the EORTC gastrointestinal tract cancer cooperative group. Ann. Surg..

[26] Takada T., Amano H., Yasuda H., Nimura Y., Matsushiro T., Kato H., Nagakawa T., Nakayama T., Study Group of Surgical Adjuvant Therapy for Carcinomas of the Pancreas and Biliary Tract (2002). Is postoperative adjuvant chemotherapy useful for gallbladder carcinoma? A phase III multicenter prospective randomized controlled trial in patients with resected pancreaticobiliary carcinoma. Cancer.

[27] Regine W.F., Winter K.A., Abrams R., Safran H., Hoffman J.P., Konski A., Benson A.B., Macdonald J.S., Rich T.A., Willett C.G. (2011). Fluorouracil-based chemoradiation with either gemcitabine or fluorouracil chemotherapy after resection of pancreatic adenocarcinoma: 5-year analysis of the U.S. Intergroup/RTOG 9704 phase III trial. Ann. Surg. Oncol..

[28] Schmidt J., Abel U., Debus J., Harig S., Hoffmann K., Herrmann T., Bartsch D., Klein J., Mansmann U., Jager D. (2012). Open-label, multicenter, randomized phase III trial of adjuvant chemoradiation plus interferon Alfa-2b versus fluorouracil and folinic acid for patients with resected pancreatic adenocarcinoma. J. Clin. Oncol..

[29] Neoptolemos J.P., Stocken D.D., Bassi C., Ghaneh P., Cunningham D., Goldstein D., Padbury R., Moore M.J., Gallinger S., Mariette C. (2010). Adjuvant chemotherapy with fluorouracil plus folinic acid vs. gemcitabine following pancreatic cancer resection: A randomized controlled trial. JAMA.

[30] Neoptolemos J.P., Palmer D.H., Ghaneh P., Psarelli E.E., Valle J.W., Halloran C.M., Faluyi O., O’Reilly D.A., Cunningham D., Wadsley J. (2017). Comparison of adjuvant gemcitabine and capecitabine with gemcitabine monotherapy in patients with resected pancreatic cancer (ESPAC-4): A multicentre, open-label, randomised, phase 3 trial. Lancet.

[31] Conroy T., Castan F., Lopez A., Turpin A., Ben Abdelghani M., Wei A.C., Mitry E., Biagi J.J., Evesque L., Artru P. (2022). Five-Year Outcomes of FOLFIRINOX vs. Gemcitabine as Adjuvant Therapy for Pancreatic Cancer: A Randomized Clinical Trial. JAMA Oncol..

[32] Hackert T., Sachsenmaier M., Hinz U., Schneider L., Michalski C.W., Springfeld C., Strobel O., Jager D., Ulrich A., Buchler M.W. (2016). Locally Advanced Pancreatic Cancer: Neoadjuvant Therapy With Folfirinox Results in Resectability in 60% of the Patients. Ann. Surg..

[33] Yachida S., Jones S., Bozic I., Antal T., Leary R., Fu B., Kamiyama M., Hruban R.H., Eshleman J.R., Nowak M.A. (2010). Distant metastasis occurs late during the genetic evolution of pancreatic cancer. Nature.

[34] Haeno H., Gonen M., Davis M.B., Herman J.M., Iacobuzio-Donahue C.A., Michor F. (2012). Computational modeling of pancreatic cancer reveals kinetics of metastasis suggesting optimum treatment strategies. Cell.

[35] Mackay T.M., Smits F.J., Roos D., Bonsing B.A., Bosscha K., Busch O.R., Creemers G.J., van Dam R.M., van Eijck C.H.J., Gerhards M.F. (2020). The risk of not receiving adjuvant chemotherapy after resection of pancreatic ductal adenocarcinoma: A nationwide analysis. HPB.

[36] Sohal D., Duong M.T., Ahmad S.A., Gandhi N., Beg M.S., Wang-Gillam A., Wade J.L., Chiorean E.G., Guthrie K.A., Lowy A.M. (2020). SWOG S1505: Results of perioperative chemotherapy (peri-op CTx) with mfolfirinox versus gemcitabine/nab-paclitaxel (Gem/nabP) for resectable pancreatic ductal adenocarcinoma (PDA). J. Clin. Oncol..

[37] Maurel J., Sanchez-Cabus S., Laquente B., Gaba L., Visa L., Fabregat J., Poves I., Rosello S., Diaz-Beveridge R., Martin-Richard M. (2018). Outcomes after neoadjuvant treatment with gemcitabine and erlotinib followed by gemcitabine-erlotinib and radiotherapy for resectable pancreatic cancer (GEMCAD 10-03 trial). Cancer Chemother. Pharmacol..

[38] Labori K.J., Bratlie S.O., Andersson B., Angelsen J.H., Biorserud C., Bjornsson B., Bringeland E.A., Elander N., Garresori H., Gronbech J.E. (2024). Neoadjuvant FOLFIRINOX versus upfront surgery for resectable pancreatic head cancer (NORPACT-1): A multicentre, randomised, phase 2 trial. Lancet Gastroenterol. Hepatol..

[39] Cecchini M., Salem R.R., Robert M., Czerniak S., Blaha O., Zelterman D., Rajaei M., Townsend J.P., Cai G., Chowdhury S. (2024). Perioperative Modified FOLFIRINOX for Resectable Pancreatic Cancer: A Nonrandomized Controlled Trial. JAMA Oncol..

[40] Bartolomucci A., Nobrega M., Ferrier T., Dickinson K., Kaorey N., Nadeau A., Castillo A., Burnier J.V. (2025). Circulating tumor DNA to monitor treatment response in solid tumors and advance precision oncology. NPJ Precis. Oncol..

[41] Chen J., Gale R.P., Hu Y., Yan W., Wang T., Zhang W. (2024). Measurable residual disease (MRD)-testing in haematological and solid cancers. Leukemia.

[42] Yaghoubi Naei V., Bordhan P., Mirakhorli F., Khorrami M., Shrestha J., Nazari H., Kulasinghe A., Ebrahimi Warkiani M. (2023). Advances in novel strategies for isolation, characterization, and analysis of CTCs and ctDNA. Ther. Adv. Med. Oncol..

[43] Ziogas D.C., Papadopoulou E., Gogas H., Sakellariou S., Felekouras E., Theocharopoulos C., Stefanou D.T., Theochari M., Boukovinas I., Matthaios D. (2023). Digging into the NGS Information from a Large-Scale South European Population with Metastatic/Unresectable Pancreatic Ductal Adenocarcinoma: A Real-World Genomic Depiction. Cancers.

[44] Gong J., Hendifar A., Gangi A., Zaghiyan K., Atkins K., Nasseri Y., Murrell Z., Figueiredo J.C., Salvy S., Haile R. (2021). Clinical Applications of Minimal Residual Disease Assessments by Tumor-Informed and Tumor-Uninformed Circulating Tumor DNA in Colorectal Cancer. Cancers.

[45] Pantel K., Alix-Panabieres C. (2025). Minimal residual disease as a target for liquid biopsy in patients with solid tumours. Nat. Rev. Clin. Oncol..

[46] Elazezy M., Joosse S.A. (2018). Techniques of using circulating tumor DNA as a liquid biopsy component in cancer management. Comput. Struct. Biotechnol. J..

[47] Watanabe K., Nakamura T., Kimura Y., Motoya M., Kojima S., Kuraya T., Murakami T., Kaneko T., Shinohara Y., Kitayama Y. (2022). Tumor-Informed Approach Improved ctDNA Detection Rate in Resected Pancreatic Cancer. Int. J. Mol. Sci..

[48] Kalluri R., Weinberg R.A. (2009). The basics of epithelial-mesenchymal transition. J. Clin. Investig..

[49] Yao D., Dai C., Peng S. (2011). Mechanism of the mesenchymal-epithelial transition and its relationship with metastatic tumor formation. Mol. Cancer Res..

[50] Eslami S.Z., Cortes-Hernandez L.E., Alix-Panabieres C. (2020). Epithelial Cell Adhesion Molecule: An Anchor to Isolate Clinically Relevant Circulating Tumor Cells. Cells.

[51] Gorges T.M., Tinhofer I., Drosch M., Rose L., Zollner T.M., Krahn T., von Ahsen O. (2012). Circulating tumour cells escape from EpCAM-based detection due to epithelial-to-mesenchymal transition. BMC Cancer.

[52] Zhao X.H., Wang Z.R., Chen C.L., Di L., Bi Z.F., Li Z.H., Liu Y.M. (2019). Molecular detection of epithelial-mesenchymal transition markers in circulating tumor cells from pancreatic cancer patients: Potential role in clinical practice. World J. Gastroenterol..

[53] Harouaka R.A., Nisic M., Zheng S.Y. (2013). Circulating tumor cell enrichment based on physical properties. J. Lab. Autom..

[54] Pietrasz D., Pecuchet N., Garlan F., Didelot A., Dubreuil O., Doat S., Imbert-Bismut F., Karoui M., Vaillant J.C., Taly V. (2017). Plasma Circulating Tumor DNA in Pancreatic Cancer Patients Is a Prognostic Marker. Clin. Cancer Res..

[55] Botta G.P., Abdelrahim M., Drengler R.L., Aushev V.N., Esmail A., Laliotis G., Brewer C.M., George G.V., Abbate S.M., Chandana S.R. (2024). Association of personalized and tumor-informed ctDNA with patient survival outcomes in pancreatic adenocarcinoma. Oncologist.

[56] Dickey E.M., Martos M.P., Yanala U., Corona A., Ezenwajiaku N., Pizzolato J., Franceschi D., Livingstone A.S., Terrero G., Hester C.A. (2025). Utility of tumor-informed circulating tumor DNA for detection of minimal residual disease after curative-intent therapy in localized pancreatic cancer. Surg. Oncol. Insight.

[57] Kitahata Y., Kawai M., Hirono S., Okada K.I., Miyazawa M., Motobayashi H., Ueno M., Hayami S., Miyamoto A., Yamaue H. (2022). Circulating Tumor DNA as a Potential Prognostic Marker in Patients with Borderline-Resectable Pancreatic Cancer Undergoing Neoadjuvant Chemotherapy Followed by Pancreatectomy. Ann. Surg. Oncol..

[58] Li P.C.-W., Winters H.D., Geng X., Wang H., Schumann A., Noel M.S., He A.R., Weinberg B.A., Marshall J., Tesfaye A.A. (2025). Real-world microscopic residual disease (MRD) monitoring with circulating tumor DNA (ctDNA) in pancreatic adenocarcinoma (PDAC). J. Clin. Oncol..

[59] Sausen M., Phallen J., Adleff V., Jones S., Leary R.J., Barrett M.T., Anagnostou V., Parpart-Li S., Murphy D., Kay Li Q. (2015). Clinical implications of genomic alterations in the tumour and circulation of pancreatic cancer patients. Nat. Commun..

[60] Alqahtani A., Alloghbi A., Coffin P., Yin C., Mukherji R., Weinberg B.A. (2023). Prognostic utility of preoperative and postoperative KRAS-mutated circulating tumor DNA (ctDNA) in resected pancreatic ductal adenocarcinoma: A systematic review and meta-analysis. Surg. Oncol..

[61] Yamaguchi T., Uemura K., Murakami Y., Kondo N., Nakagawa N., Okada K., Seo S., Hiyama E., Takahashi S., Sueda T. (2021). Clinical Implications of Pre- and Postoperative Circulating Tumor DNA in Patients with Resected Pancreatic Ductal Adenocarcinoma. Ann. Surg. Oncol..

[62] Vidal L., Pando E., Blanco L., Fabregat-Franco C., Castet F., Sierra A., Macarulla T., Balsells J., Charco R., Vivancos A. (2023). Liquid biopsy after resection of pancreatic adenocarcinoma and its relation to oncological outcomes. Systematic review and meta-analysis. Cancer Treat. Rev..

[63] Maulat C., Canivet C., Cabarrou B., Pradines A., Selves J., Casanova A., Doussine A., Hanoun N., Cuellar E., Boulard P. (2024). Prognostic impact of circulating tumor DNA detection in portal and peripheral blood in resected pancreatic ductal adenocarcinoma patients. Sci. Rep..

[64] Avula L.R., Hagerty B., Alewine C. (2020). Molecular mediators of peritoneal metastasis in pancreatic cancer. Cancer Metastasis Rev..

[65] Leick K.M., Tomanek-Chalkley A., Coleman K.L., Chan C.H.F. (2023). Peritoneal Cell-Free Tumor DNA is a Biomarker of Locoregional and Peritoneal Recurrence in Resected Pancreatic Ductal Adenocarcinomas. Ann. Surg. Oncol..

[66] Nakano Y., Kitago M., Matsuda S., Nakamura Y., Fujita Y., Imai S., Shinoda M., Yagi H., Abe Y., Hibi T. (2018). KRAS mutations in cell-free DNA from preoperative and postoperative sera as a pancreatic cancer marker: A retrospective study. Br. J. Cancer.

[67] Lee B., Lipton L., Cohen J., Tie J., Javed A.A., Li L., Goldstein D., Burge M., Cooray P., Nagrial A. (2019). Circulating tumor DNA as a potential marker of adjuvant chemotherapy benefit following surgery for localized pancreatic cancer. Ann. Oncol..

[68] Groot V.P., Mosier S., Javed A.A., Teinor J.A., Gemenetzis G., Ding D., Haley L.M., Yu J., Burkhart R.A., Hasanain A. (2019). Circulating Tumor DNA as a Clinical Test in Resected Pancreatic Cancer. Clin. Cancer Res..

[69] Hata T., Mizuma M., Motoi F., Ohtsuka H., Nakagawa K., Morikawa T., Unno M. (2023). Prognostic impact of postoperative circulating tumor DNA as a molecular minimal residual disease marker in patients with pancreatic cancer undergoing surgical resection. J. Hepato-Biliary-Pancreat. Sci..

[70] Tie J., Wang Y., Lo S.N., Lahouel K., Cohen J.D., Wong R., Shapiro J.D., Harris S.J., Khattak A., Burge M.E. (2025). Circulating tumor DNA analysis guiding adjuvant therapy in stage II colon cancer: 5-year outcomes of the randomized DYNAMIC trial. Nat. Med..

[71] Tie J., Cohen J.D., Lahouel K., Lo S.N., Wang Y., Kosmider S., Wong R., Shapiro J., Lee M., Harris S. (2022). Circulating Tumor DNA Analysis Guiding Adjuvant Therapy in Stage II Colon Cancer. N. Engl. J. Med..

[72] Verschoor N., Bos M.K., Oomen-de Hoop E., Martens J.W.M., Sleijfer S., Jager A., Beije N. (2024). A review of trials investigating ctDNA-guided adjuvant treatment of solid tumors: The importance of trial design. Eur. J. Cancer.

[73] Lee B., Tie J., Wang Y., Cohen J.D., Shapiro J.D., Wong R., Aghmesheh M., Kiberu A.D., Francesconi A., Burge M.E. (2024). The potential role of serial circulating tumor DNA (ctDNA) testing after upfront surgery to guide adjuvant chemotherapy for early stage pancreatic cancer: The AGITG DYNAMIC-Pancreas trial. J. Clin. Oncol..

[74] Pascual J., Attard G., Bidard F.C., Curigliano G., De Mattos-Arruda L., Diehn M., Italiano A., Lindberg J., Merker J.D., Montagut C. (2022). ESMO recommendations on the use of circulating tumour DNA assays for patients with cancer: A report from the ESMO Precision Medicine Working Group. Ann. Oncol..

[75] Bettegowda C., Sausen M., Leary R.J., Kinde I., Wang Y., Agrawal N., Bartlett B.R., Wang H., Luber B., Alani R.M. (2014). Detection of circulating tumor DNA in early- and late-stage human malignancies. Sci. Transl. Med..

[76] Henriksen T.V., Demuth C., Frydendahl A., Nors J., Nesic M., Rasmussen M.H., Reinert T., Larsen O.H., Jaensch C., Love U.S. (2024). Unraveling the potential clinical utility of circulating tumor DNA detection in colorectal cancer-evaluation in a nationwide Danish cohort. Ann. Oncol..

[77] Umemoto K., Sunakawa Y., Ueno M., Furukawa M., Mizuno N., Sudo K., Kawamoto Y., Kajiwara T., Ohtsubo K., Okano N. (2023). Clinical significance of circulating-tumour DNA analysis by metastatic sites in pancreatic cancer. Br. J. Cancer.

[78] Bai H., Wang Z., Chen K., Zhao J., Lee J.J., Wang S., Zhou Q., Zhuo M., Mao L., An T. (2012). Influence of chemotherapy on EGFR mutation status among patients with non-small-cell lung cancer. J. Clin. Oncol..

[79] Evan T., Wang V.M., Behrens A. (2022). The roles of intratumour heterogeneity in the biology and treatment of pancreatic ductal adenocarcinoma. Oncogene.

[80] Shah D., Wells A., Cox M., Dawravoo K., Abad J., D’Souza A., Suh G., Bayer R., Chaudhry S., Zhang Q. (2025). Prospective Evaluation of Circulating Tumor DNA Using Next-generation Sequencing as a Biomarker During Neoadjuvant Chemotherapy in Localized Pancreatic Cancer. Ann. Surg..

[81] Bryant J.M., Ruffolo L., Soares K., Hoffe S., Lowy A.M. (2025). Evolving concepts in adjuvant/neoadjuvant therapy for resectable pancreas cancer. J. Clin. Investig..

[82] Vitello D.J., Shah D., Wells A., Masnyk L., Cox M., Janczewski L.M., Abad J., Dawravoo K., D’Souza A., Suh G. (2024). Mutant KRAS in Circulating Tumor DNA as a Biomarker in Localized Pancreatic Cancer in Patients Treated with Neoadjuvant Chemotherapy. Ann. Surg..

[83] Toledano-Fonseca M., Brozos-Vazquez E., Garcia-Ortiz M.V., Costa-Fraga N., Diaz-Lagares A., Rodriguez-Ariza A., Lopez-Lopez R., Aranda E. (2025). Unveilling the potential of liquid biopsy in pancreatic cancer for molecular diagnosis and treatment guidance. Crit. Rev. Oncol. Hematol..

[84] Valle J.W., Palmer D., Jackson R., Cox T., Neoptolemos J.P., Ghaneh P., Rawcliffe C.L., Bassi C., Stocken D.D., Cunningham D. (2014). Optimal duration and timing of adjuvant chemotherapy after definitive surgery for ductal adenocarcinoma of the pancreas: Ongoing lessons from the ESPAC-3 study. J. Clin. Oncol..

[85] Ceelen W., Demuytere J., de Hingh I. (2021). Hyperthermic Intraperitoneal Chemotherapy: A Critical Review. Cancers.

[86] Sugarbaker P.H., Stuart O.A. (2021). Intraperitoneal gemcitabine chemotherapy is safe for patients with resected pancreatic cancer: Final clinical and pharmacologic data from a phase II protocol and recommended future directions. J. Gastrointest. Oncol..

[87] Tentes A.K. (2021). Hyperthermic intra-operative intraperitoneal chemotherapy as an adjuvant to pancreatic cancer resection. J. Gastrointest. Oncol..

[88] Kasi A., Diaz F., Al-Rajabi R.M.d.T., Baranda J.C., Carroll E., Belcher C., Olyaee M., Rastogi A., Schmitt T., Kumer S. (2023). Targeting minimal residual disease (MRD) in resected RAS mutated pancreatic cancer with vaccine TG01/QS-21 +/- PD-1 inhibitor, balstilimab: A randomized phase II study (TESLA). J. Clin. Oncol..

[89] Pinson J., Henriques J., Beaussire L., Sarafan-Vasseur N., Sa Cunha A., Bachet J.B., Vernerey D., Di Fiore F., Schwarz L., group P.P. (2024). New Biomarkers to Define a Biological Borderline Situation for Pancreatic Adenocarcinoma: Results of an Ancillary Study of the PANACHE01-PRODIGE48 Trial. Ann. Surg..

[90] O’Kane G.M., Grunwald B.T., Jang G.H., Masoomian M., Picardo S., Grant R.C., Denroche R.E., Zhang A., Wang Y., Lam B. (2020). GATA6 Expression Distinguishes Classical and Basal-like Subtypes in Advanced Pancreatic Cancer. Clin. Cancer Res..

[91] Kato A., Naiki-Ito A., Naitoh I., Hayashi K., Nakazawa T., Shimizu S., Nishi Y., Okumura F., Inoue T., Takada H. (2018). The absence of class III beta-tubulin is predictive of a favorable response to nab-paclitaxel and gemcitabine in patients with unresectable pancreatic ductal adenocarcinoma. Hum. Pathol..

[92] Sahin T.K., Isik A., Guven D.C., Ceylan F., Babaoglu B., Akyol A., Yalcin S., Dizdar O. (2024). The prognostic and predictive role of class III beta-Tubulin and hENT1 expression in patients with advanced pancreatic ductal adenocarcinoma. Pancreatology.

[93] Theocharopoulos C., Davakis S., Ziogas D.C., Theocharopoulos A., Foteinou D., Mylonakis A., Katsaros I., Gogas H., Charalabopoulos A. (2024). Deep Learning for Image Analysis in the Diagnosis and Management of Esophageal Cancer. Cancers.

[94] Alum E.U., Ugwu O.P.-C. (2025). Artificial intelligence in personalized medicine: Transforming diagnosis and treatment. Discov. Appl. Sci..

[95] Dixon D., Sattar H., Moros N., Kesireddy S.R., Ahsan H., Lakkimsetti M., Fatima M., Doshi D., Sadhu K., Junaid Hassan M. (2024). Unveiling the Influence of AI Predictive Analytics on Patient Outcomes: A Comprehensive Narrative Review. Cureus.

[96] Abbosh C., Birkbak N.J., Swanton C. (2018). Early stage NSCLC—Challenges to implementing ctDNA-based screening and MRD detection. Nat. Rev. Clin. Oncol..

[97] Shin S.H., Cha S., Lee H.Y., Shin S.H., Kim Y.J., Park D., Han K.Y., Oh Y.J., Park W.Y., Ahn M.J. (2024). Machine learning model for circulating tumor DNA detection in chronic obstructive pulmonary disease patients with lung cancer. Transl. Lung Cancer Res..

[98] Widman A.J., Shah M., Frydendahl A., Halmos D., Khamnei C.C., Ogaard N., Rajagopalan S., Arora A., Deshpande A., Hooper W.F. (2024). Ultrasensitive plasma-based monitoring of tumor burden using machine-learning-guided signal enrichment. Nat. Med..

[99] Shen C., Rawal S., Brown R., Zhou H., Agarwal A., Watson M.A., Cote R.J., Yang C. (2023). Automatic detection of circulating tumor cells and cancer associated fibroblasts using deep learning. Sci. Rep..

[100] Wu J., Wang H., Nie Y., Wang Y., He W., Wang G., Li Z., Chen J., Xu W. (2025). CTCNet: A fine-grained classification network for fluorescence images of circulating tumor cells. Med. Biol. Eng. Comput..

[101] Yang Y., Wang Z., Hao T., Ye M., Li J., Zhang Q., Guo Z. (2024). Deep Learning-Assisted Assessing of Single Circulating Tumor Cell Viability via Cellular Morphology. Anal. Chem..

